# An Entropy-Based Car Failure Detection Method Based on Data Acquisition Pipeline

**DOI:** 10.3390/e21040426

**Published:** 2019-04-22

**Authors:** Bartosz Kowalik, Marcin Szpyrka

**Affiliations:** Department of Applied Computer Science, Faculty of Electrical Engineering, Automatics, Computer Science and Biomedical Engineering, AGH University of Science and Technology, al. Mickiewicza 30, 30-059 Kraków, Poland

**Keywords:** car failure detection, entropy, data mining

## Abstract

Modern cars are equipped with plenty of electronic devices called Electronic Control Units (ECU). ECUs collect diagnostic data from a car’s components such as the engine, brakes etc. These data are then processed, and the appropriate information is communicated to the driver. From the point of view of safety of the driver and the passengers, the information about the car faults is vital. Regardless of the development of on-board computers, only a small amount of information is passed on to the driver. With the data mining approach, it is possible to obtain much more information from the data than it is provided by standard car equipment. This paper describes the environment built by the authors for data collection from ECUs. The collected data have been processed using parameterized entropies and data mining algorithms. Finally, we built a classifier able to detect a malfunctioning thermostat even if the car equipment does not indicate it.

## 1. Introduction

Car reliability degrades over time. The driven distance is important, but car parts wear out and deteriorate even if a car stands unused for months. Rust builds up on brake rotors, rubber parts rot and leak etc. Conditions in which the vehicle is being used are also vital. Driving in the city differs a lot from driving on the highways. In the city a car is driven on different RPMs; it starts and stops a lot. On the other hand, on the highway a car is forced to maintain constant speed for a considerable amount of time. People rely on cars and cars break from time to time.

Modern cars equipped with plenty of Electronic Control Units (ECU) are able to detect a fault and pass on the relevant information to the driver. Unfortunately, in most cases the message is reduced to a warning light on the dashboard, and only an authorized car service is able to diagnose the fault based on error codes. Thus, a question can be asked: if we had access to data read by sensors, could we diagnose the situation ourselves? And even more, what additional knowledge about the car’s work can be obtained from such data? For example, can we assess the driver’s driving style?

This article is meant to provide a partial answer to these questions. It describes a data acquisition system that collects data from car’s ECUs and prepares them for further exploration with the data mining techniques. The system was built with a minimum financial effort. Its components include an Android based smartphone, Torque PRO application, and Akka HTTP framework. The data acquisition system captures live stream of the data from a car, stores it and does not influence the driver. The prototype versions of our system were presented in two conference papers [1,2]. The most important step involved decoding the Torque protocol.

The presented system was successfully used to collect real data from Hyundai i30 vehicle. The data set consists of 44 h of car driving. By collecting the first data set, we were able to obtain the data from a car with a malfunctioning thermostat and after the thermostat had been repaired. We then used this data to build a classifier to detect the analyzed sample points in a malfunctioning thermostat. As it turned out, the use of parameterized entropy significantly influenced the quality of the obtained results.

This paper is organized as follows: Section 2 presents related works. The data acquisition system is presented in Section 3. Section 4 deals with a short introduction to parameterized entropies. The collected data set is described in Section 5. Some results of the data exploration are presented in Section 6. A short summary is given in the final section.

## 2. Related Work

One of the analyzed existing solutions is based on both machine learning and entropy [3]. Designed solution uses permutation entropy as a feature in a machine learning model. Entropy has been calculated for normalized samples. Pipeline includes both failure detection and classification which is even a step further, but classification requires more complex processing. What is noticeable is that researchers used vibration signals to determine behavior of the rolling element.

The paper [4] describes an engine diagnosis based on acoustic signals that it emits. Because the analyzed data set contained fairly complex signal the authors used different types of Artificial Neural Networks to detect the fault type. Multiple samples were recorded for each engine ensuring first that it operated normally. For proper fault detection it is required to obtain sound samples on certain engine revolutions.

A very similar method to [3,4], presented in the paper [5], uses emitted signal, in this particular example—vibrations. Unlike described earlier, only statistical measures were used to diagnose the examined part—in this case planetary gearbox.

The approach described in [6] uses an expert system to diagnose potential faults of a single model and make. The developed expert system is based on Bayesian approach. The main difference is that a car user needs to select preset conditions by selecting applying options. Next, the application calculates with amount of probability what is the fault reason. Another expert system described here [7] also uses an expert system to diagnose subset of potential faults.

Multiple Bayesian neural networks were applied to increase diagnostic accuracy of ground-source heat pump system in [8]. They are based on sensor data and human information. Each network consists of two layers. The first layer is responsible for detecting faults, second one detects fault symptoms. Human based information improved overall accuracy of this solution.

Object-oriented Bayesian networks (OOBNs) were used in real-time fault diagnosis [9]. Introduced object-oriented approach allowed to reduce overall complexity. When modelling OOBN a first step is an off-line feed with an expert knowledge and sensor historical data. Than in an on-line phase expert knowledge is used along with real sensor data. Those information combined result in fault diagnosis.

In general, the solutions fall into two groups. The diagnosis requires either special tools [3,4], or specialized knowledge or expertise [6,7]. In consequence, those techniques are hardly applicable by day-to-day drivers.

This paper describes a system which acquires driving data from a car and is able to process information in real time. The whole process includes both a live analysis and raw data persistence. What is more, the analytical part uses machine learning algorithm to diagnose thermostat issues. As a result, multiple models were developed, one of which is entropy based. One of the major challenges was to find proper parameters for parameterized entropy. The synergy of machine learning and parameterized entropy gave a compact model.

Main contributions can be summarized as follow:

- An online system was significantly improved to collect and analyze live data from a driven car.
- Using parameterized entropy as a feature in a machine learning model resulted in a compact and accurate model.
- It was possible to diagnose not obvious car faults using cheap and widely available equipment; no specialized tool was used in this research.
- We build a system that does not require neither specialized knowledge nor prior preparations to diagnose failures.

## 3. Data Acquisition

The data for the presented research were collected from Hyundai i30 CW manufactured in 2009 and equipped with ODB2 diagnostic interface. The amount of data produced by the car requires an automated processing pipeline. Such data acquisition system was developed as part of this research. The system needed to work on-line while the car was going and could not require interaction with a driver.

The diagnostic data are collected by an ODB2 ELM327 microchip. The ODB2 reader connects to a Xiaomi Redmi 4X smartphone equipped with Torque Pro Android application via Bluetooth. The Torque application collects live data from the car. The captured interval is configurable and based on the experiments it was set to 1 s.

Torque Pro application, apart from saving the collected data to a local file on the smartphone, allows the user to set custom HTTP URL where the data are sent. It sends the data over the HTTP encoded in a query string. In order to find out what parameters are being sent, configuration file from the application were reverse-engineered. Identifiers along with labels are stored in the file named torqueConf.dat. Example data is presented below:

- log_min46 = 0.0 – minimal value,
- log_max46 = 100.0 – maximal value,
- log_pid46 = 16716401 – identifier number,
- log_fullName46 = Fuel used (trip) – description,
- log_unit46 = l – unit.

The application stores parameter IDs in a decimal number format but sends them in hexadecimal. What is more, it maps internally between its own parameter ordering and external IDs. The above-mentioned parameter 46 with ID (decimal) 16716401 has hexadecimal FF5203. An example URI sent by the application is presented in Listing 1 (Line breaking was used to make the text more readable):
**Listing 1.**Example URI?eml= bartekviper@gmail .com&kff1222 =0.47763458&v=8&kff1221 =0.7290559&session =1529270311928&kff1239 =4.5509996&id =80 c9065bff55006e9f5f3d4f8d9456ae&kff1269 =49.0& time =1529271850358&kff1005 =20.00668569&kff1005 =20.00668569&kff1006 =50.08636538&kff1006 =50.08636538&kff123b =0.0&kff1001 =0.0&kff1203 =0.0&kff1007 =0.0&kff5202 =5.5996585&kff129a =61.0&kff5203 =17.85786&k2d =99.21875&kff1201 =0.0&k33 =98.0&kff5201 =15.818244&kb =98.0&kff1238 =13.0&k23 =8560.0&k42 =13.212&kff1267 =109.728&k4 =0.0&kff1268 =438.995&kc =0.0&kff1266 =548.0&k21 =0.0&kff1223 = -0.030030591&k31 =65535.0&kff1220 =0.2738867&kff126b =2.0411227&kff126a =33.774616&k5 =95.0&kff1204 =5.892079&kff1202 =0.0&k10 =3.52&kff1206 =7.2493286&kff1001 =0.0&kff1010 =268.0&kd =0.0&kff1271 =1.289121&kff1237 =0.0&kff125d =1.286436&kff123a =17.0&kff125a =21.4406&k2c =4.7058825&kff1272 =22.889725&k46 =28.0&kff1208 =13.794094&kf =39.0


A performed request contains standard OBD data, such as engine coolant temperature and data calculated by Torque Pro, for example, the fuel used. Meta information is also sent and it contains:

- eml—registered email address,
- v—protocol version,
- session—when measurement started in milliseconds since 1 January 1970 UTC,
- id—UUDI session identifier with stripped “-”,
- time—measurement time in milliseconds since 1 January 1970 UTC.

The server is capable of processing data with high throughput. It allows for creation of multiple connections for different test drives. Torque Pro sends standard OBD2 PIDs (Parameter IDs) along with the proprietary ones.

The server saves collected data into InfluxD—a time series database. It is designed to store and query time-oriented data without delays. This feature is important from server’s perspective. Each measured attribute is stored in a separate table with a tag assigned to it. Tags allow for identification of test drives.

Data is not only saved to a database but also published on queues. There is one queue per each measured attribute. The server publishes messages in JSON (JavaScript Object Notation) format. Ther can be multiple independent subscribers for each queue so data can be processed in real time.

## 4. Entropy

This section provides theoretic fundamentals of entropy. It starts with a brief overview of Shannon entropy and then the parameterized generalizations are presented.

Entropy as a measure of disorder has its origin in thermodynamic. The concept was proposed in the early 1850s by Clausius [10]. Shannon [11] adopted entropy to information theory in 1948. In information theory, the concept is used to measure the uncertainty of a random variable – the greater entropy, the more random variable. Let *X* be a random variable that can take values $\left\{x_{1}\ldots x_{n}\right\}$, and $p(x_{i})$ is the *probability mass function* of outcome $x_{i}$. The Shannon entropy for variable *X* is defined as:

(1) $$H_{s}\left(X\right)=\sum_{i=1}^{n}p\left(x_{i}\right)\log_{a}\frac{1}{p(x_{i})}$$

Depending on the value of *a* parameter, different units can be used: bits (a=2), nats (a=e) or hurtleys (a=10). For more details about the Shannon entropy see [12,13].

The Shannon entropy assumes a trade-off between contributions from the main mass of the distribution and the tail [14]. In order to control the trade-off explicitly, we must use a generalization of the Shannon entropy. Two such generalizations were proposed by Renyi [15] and Tsallis [16] respectively. The Renyi entropy is defined as:

(2) $$H_{R\alpha }\left(X\right)=\frac{1}{1-\alpha }\log_{a}\left(\sum_{i=1}^{n}p{\left(x_{i}\right)}^{\alpha }\right)$$

while the Tsallis entropy is defined as:

(3) $$H_{T\alpha }\left(X\right)=\frac{1}{1-\alpha }\left(\sum_{i=1}^{n}p{\left(x_{i}\right)}^{\alpha }-1\right)$$

If the parameter denoted as α has a positive value, it exposes the main mass (the concentration of events that occur often), whereas, if the value is negative, it exposes the tail (the dispersion caused by rare events). Both entropies converge to Shannon entropy for α→1. A more detailed comparison of all the above-mentioned entropies can be found in [17].

Let us consider the data sample presented in Table 1. The table contains 50 values drawn from the set presented in Table 2. Probability distribution for these values is also shown in Table 2.

**Figure 1:**
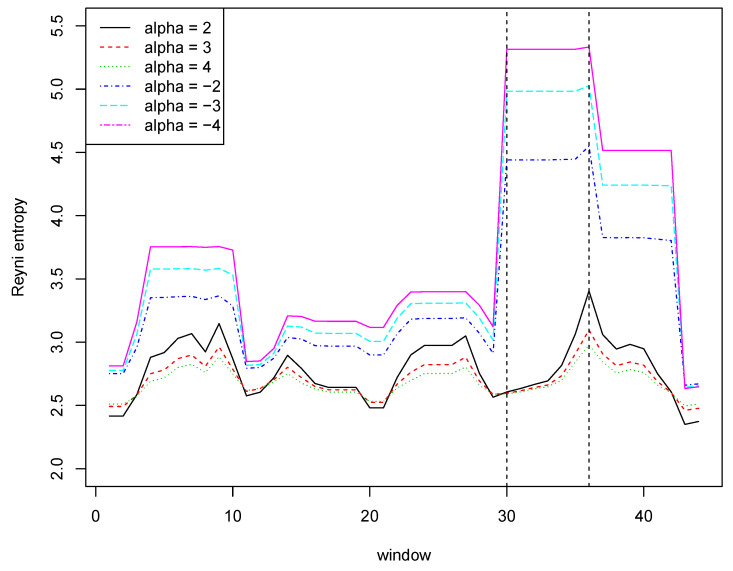
Reyni entropies for the data from Table 1.

Now, let us examine what is the impact of a rare event on the Renyi entropy with different values of the α parameter. We used a sliding window with a length of 7, i.e., the first window contains values with indices ranging from 1 to 7, the second window contains values with indices ranging from 2 to 8, etc. Figure 1 shows Reyni entropy values for six different values of the α parameter. It is worth paying attention to the entropy values for window number 30 and six consecutive windows. These windows contain the measurement number 36 containing a value with a very low probability (rarely occurring). This fact is illustrated very well by a significant increase in the value of entropy with negative coefficients.

## 5. Dataset

In order to verify if entropy-based approach is suitable to detect a car failure we used the data acquisition system described in Section 3 to collect data from the mentioned Hyundai i30 CW. We collected data corresponding to a total of 44 h of driving. The data are stored in text files using *Comma Separated Values* format (CSV). Each file corresponds to a single drive. The first line of such a file contains header (attributes names) and each of the other lines contains one data record. A snippet of one of the files is shown in Listing 2. The data are displayed in columns instead of rows, to make them more readable.
**Listing 2.** Example log file.GPS Time,Wed Dec 27 20:58:49 GMT +01:00 2017Device Time,27-gru-2017 20:58:48.388Longitude,20.4570165Latitude,50.80177848GPS Speed (Meters/second),8.24Horizontal Dilution of Precision,3.0Altitude,318.0Bearing,109.7G(x),0.86602783G(y),7.83947754G(z),4.78611755G(calibrated),0.00805598EGR Error(%),-Barometric pressure (from vehicle)(psi),-Intake Manifold Pressure(psi),15.37400055Fuel Rail Pressure(psi),-Run time since engine start(s),-Trip time(whilst stationary)(s),0Trip time(whilst moving)(s),0Trip Time(Since journey start)(s),0GPS Bearing(°),109.69999695Timing Advance(°),-Litres Per 100 Kilometer(Instant)(l/100km),-Horsepower (At the wheels)(hp),-Engine kW (At the wheels)(kW),-Torque(Nm),-Voltage (OBD Adapter)(V),-Voltage (Control Module)(V),-Engine Load(%),-Engine RPM(rpm),-Distance travelled with MIL/CEL lit(km),-Distance travelled since codes cleared(km),-Percentage of City driving(%),100Percentage of Highway driving(%),0Percentage of Idle driving(%),0Trip Distance(km),-Trip distance (stored in vehicle profile)(km), 491.32943726Mass Air Flow Rate(g/s),17.04999924Speed (OBD)(km/h),-EGR Commanded(%),-Ambient air temp(°C),-Intake Air Temperature(°C),-Engine Coolant Temperature(°C),68Turbo Boost & Vacuum Gauge(psi),0.67400074Trip average KPL(kpl),-Trip average Litres/100 KM(l/100 km)-

Table 3 consists of all measured attributes with a short description. It is important to note that either it was not possible to measure all of them, or their meaning differs based on the engine type—either it is diesel or a gasoline engine. For example the *timing advance* is used in gasoline engines to prevent the knock effect, by adjusting when a spark plug should give a spark. In diesel engines a knock effect can also occur, but fuel injectors are used to eliminate this undesirable effect.

The collected data contains measurements performed on the car with a malfunctioning thermostat. It remained half-open, causing the engine temperature to drop below the operating temperature. It also led to cabin heating loss. For validation purposes, after replacing thermostat, new data were collected describing how normally operating thermostat influences the engine coolant temperature.

Broken thermostat data set consists of 25 files. This amounts to around 16 driving hours of raw data. The second data set with the working thermostat consists of 49 files which equal to around 27 driving hours.

**Figure 2:**
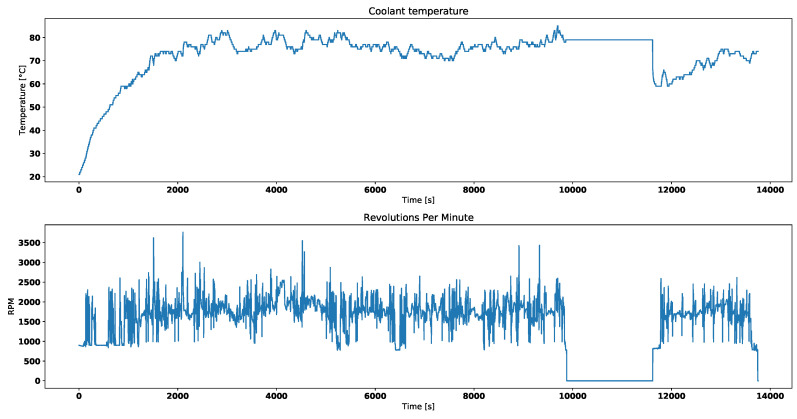
Engine coolant temperature and engine RPM for broken thermostat.

The first step involved preprocessing phase to prepare data for further analysis. It is assumed that for diagnostics purposes, engine must be running in order to produce heat and cause thermostat to open and close. All data rows where engine revolutions per minute were indicating that engine had not been running were discarded. Thus samples between around 10,000th and 12,000th second in Figure 2 were discarder and consequently in every other dataset. However, it does not matter if engine was idling or not because engine coolant flow is caused by water pump.

Figure 2 presents the broken thermostat behavior. There are both engine coolant temperature and engine RPM measurements. The engine is running when RPM readouts are above 0 and around 1000. When it is stopped coolant does not circulate, thus sensor reads local temperature. Around 12,000th second, since test drive had begun, engine was started again and temperature read dropped, because the engine got colder. The engine water pump, which forces coolant to circulate, is propelled by either timing mechanism or fan belt.

**Figure 3:**
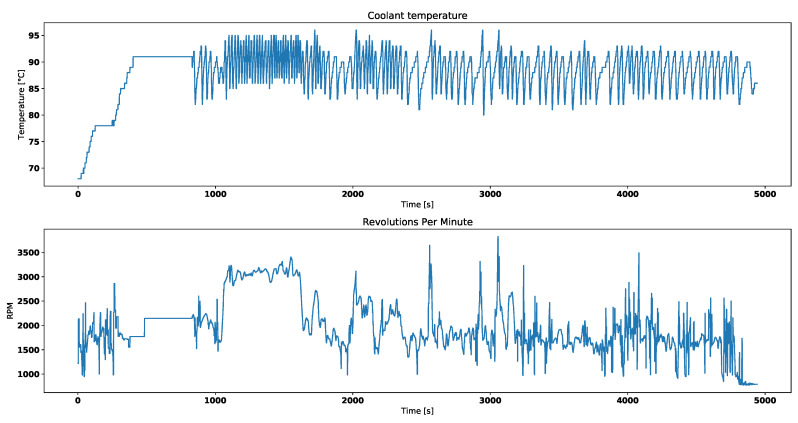
Engine coolant temperature and engine RPM for working thermostat.

In comparison Figure 3 sets the engine coolant temperature together with the engine RPM for a working thermostat. Before test drive started it already had some residual heat. Before engine reached its operating temperature, thermostat remained closed. This is why the engine is required to run.

After completing the first step, all data was divided into batches. Each of them contains 600 samples which equal 10 min of data. Last but not least, those batches were enriched with calculated entropies. Within batches, a sliding window of 10 samples was selected which amounting 10 s. Inside selected time window both Tsallis and Renyi entropies were calculated for both negative and positive values of the α parameter. Entropies rely on reference probability calculated based on a reference model. We used the sample shown in Figure 3 to determine the probabilities of individual temperature values. For values not present in the sample, it is assumed that the probability is equal to the least of the calculated ones. The calculated probabilities are given in Table 4. The smallest value is equal to 0.004653044709690472.

After many experiments following α parameters for Renyi and Tsallis entropies were selected and are presented in Table 5.

**Figure 4:**
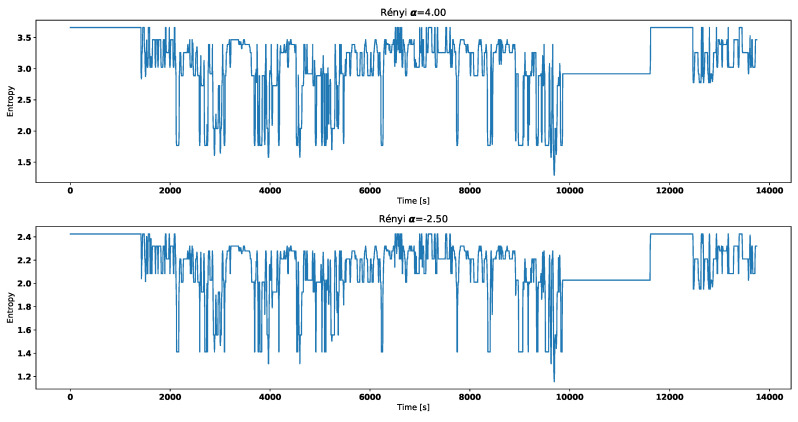
Renyi entropy for broken thermostat.

**Figure 5:**
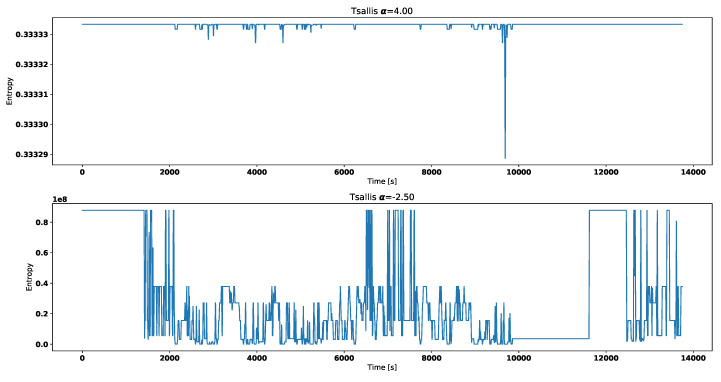
Tsallis entropy for broken thermostat.

Both Figure 4 and Figure 5 correspond with temperature data presented in Figure 2.

**Figure 6:**
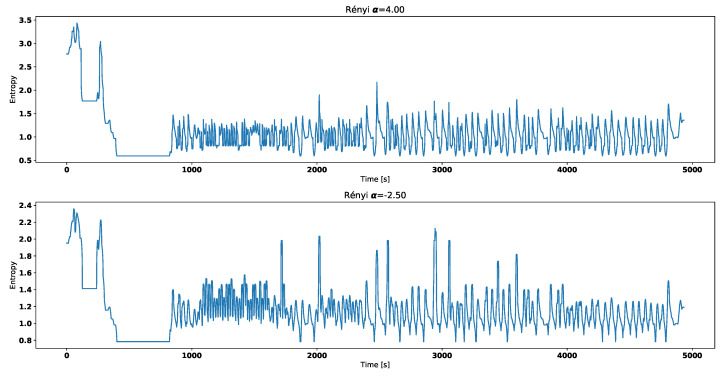
Renyi entropy for working thermostat.

**Figure 7:**
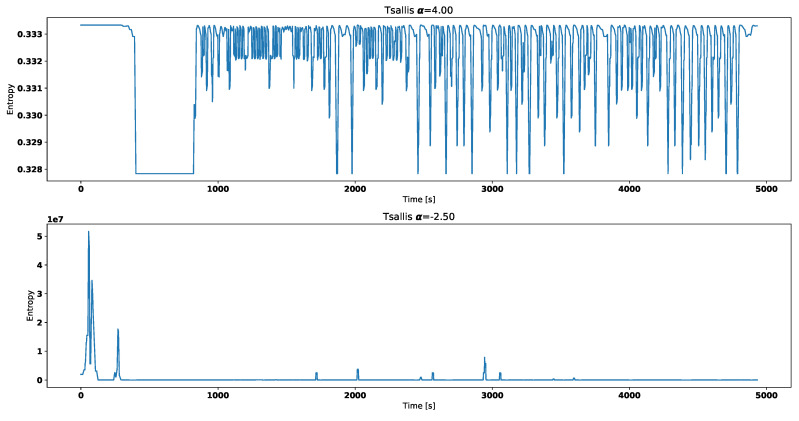
Tsallis entropy for working thermostat.

Figure 6 and Figure 7 correspond with temperature data presented in Figure 3.

Visual analysis of both Figure 2 and Figure 3 allows to distinguish working thermostat from broken one. A thermostat which operates correctly should maintain the engine temperature in given temperature range, as show in Figure 3. A broken thermostat will either leak or not open. In this case, it leaked and caused the engine to cool excessively. Undesirable behavior can be observed when analyzing entropies’ values. It is very noticeable when looking at Tsallis entropies. For working thermostat and negative α, calculated value is almost always equal to 0 with some local aberrations—see Figure 7. The same can be noticed for positive α when analyzing broken thermostat—see Figure 5. Renji entropy plots also differ for working and broken thermostat. But differences for both positive and negative α are not that significant as with Tsallis entropy.

## 6. Machine Learning Approach

It is possible to distinguish between broken and working thermostat characteristics on graphs. One of the goals was to create an automated system which would support car diagnosis. It is especially important because a standard diagnosis procedure is based on experiments which can possibly do further damage to the engine if the thermostat do not open in the right moment. What is also important is that such systems should spot advancing wear.

There are two ways of diagnosing. First is based on threshold, second on machine learning algorithm. Threshold has major disadvantage. It assumes that there is a certain temperature range in which thermostat should operate. Any temperature outside a given range is a sign of fault. More advanced solution uses machine learning algorithms. Major advantage of ML over standard algorithm is model’s ability to generalize. It also requires samples, which are diverse.

In case of this study classification decision tree was used. It is a supervised machine learning algorithm which is designed to, based on provided features (attributes), detect which sample belongs to which group. In this experiment there were two groups: working and broken. Machine learning process consists of two phases: training and testing. The entire data set was divided into two subsets corresponding to each mentioned phase. Because training is more important than testing, testing only validates quality of model, it contains 80% of all available samples.

Following experiments with different features were conducted:

1. Model A – temperature only,
2. Model B – Tsallis entropy,
3. Model C – Renji entropy, Tsallis entropy, mean and median temperature.

For model’s quality verification following measures were done:

- feature importance – in what percentage given feature has impact on a result,
- mean squared error (MSE) – measure which indicates how accurate is the model,
- model’s score – according to documentation it is mean error rate.

There were three experiments conducted with different parameters described in Table 6. As a result of each experiment different models were created. There are based on machine learning algorithm called decision tree. This algorithm labeled data from a test set as *working* or *broken*. For all models, values are labeled based on to which leaf they belong to.

**Figure 8:**
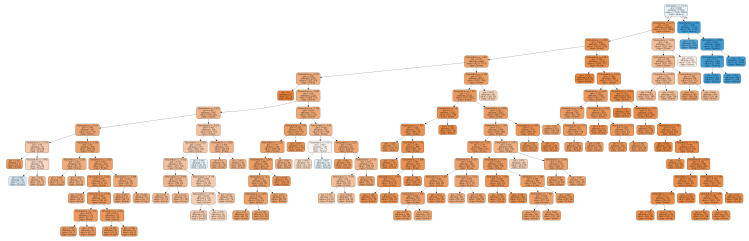
Decision tree based on temperature value.

Decision tree created for model A, presented in Figure 8, has a broad structure. It would have less nodes only if the subtree on the left do not contain nodes labeled as working. It scored 86.8% accuracy and MSE equal to 13.2%.

**Figure 9:**
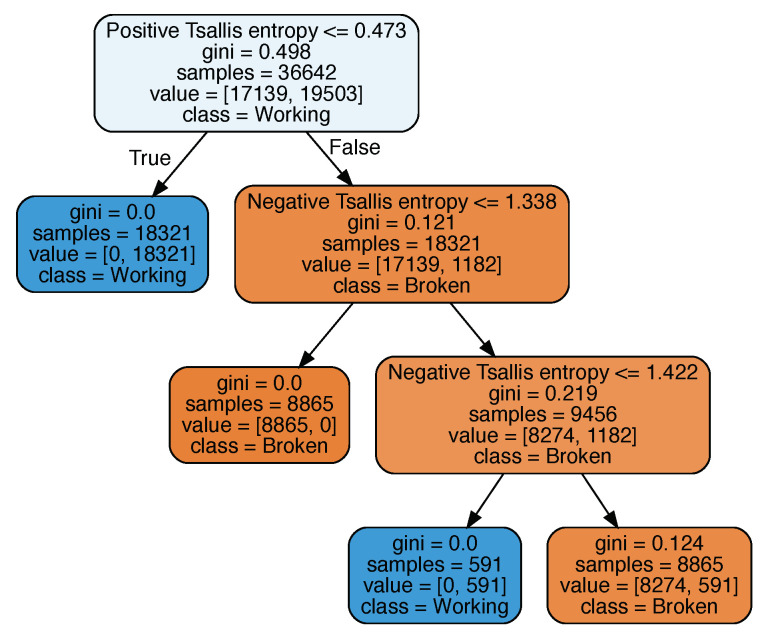
Decision tree based on Tsallis entropy.

Model B (see Figure 9) was based solely on Tsallis entropy values both for positive and negative α. It is the least complicated when compared to all the performed experiments. It also confirms the observed regularity that Tsallis entropy is sufficient to determine thermostat condition. The model scores 88.9% and MSE is equal to 11.1%. Feature importance shows that positive α has 93.5% of impact on a model’s prediction while negative α only 6.5%.

The last developed model C is based on both Tsallis and Renji entropies for positive and negative α. For the sake of this experiment mean and median temperatures are also used. This makes it too detailed and the number of contained nodes make it impossible to visualize. Renji entropy is irrelevant, because both values calculated for positive and negative α have significance of 0.0%. Tsallis entropy has performed similar to model B reaching significance of 92.5% for positive α and 5.6% for negative α. Mean temperature is 1.8% relevant to overall performance and median has less than 0.1% importance. Model scored 88.8% of correctness.

## 7. Conclusions

Entropies allow the creation of a concise decision tree model. In particular, Tsallis entropy which alone allowed the creation of model B with accuracy of 88.9%. Although, all models’ scores vary only by a few percent, model B is the least complicated. Thus, it is most generic of all.

For an experienced car mechanic investigating a thermostat problem is a simple job. Lack of cabin heating and significant engine temperature drop are noticeable symptoms for thermostat broken in an open position. On the other hand, when thermostat is not able to open itself the engine overheats and damages, for example, the engine block.

It is believed that self-driving cars will become popular on public roads within the next couple of years. A lot of effort is put into artificial intelligence which drives those cars. It seems that undisturbed operation has been put in second place. One of authors’ goals is to provide reliable solution which improves day to day car usage and will have impact on self-driving cars in the future.

From drivers’ perspective solution should be intuitive and cheap. The developed system meets both criteria although it has limited functionality for now. Mentioned criteria are crucial from the adoption standpoint. Expensive or unintuitive solutions are unacceptable for the end user. In addition, client-server solutions are universal as smartphones are responsible for collecting data and sending them to the server. Battery life is not an issue here because it is assumed that device will be used only for diagnostic purposes or will be connected to a charger.

Future works include developing a dedicated device powered by OBD2 plug. Such a device could be kept hidden in a car and work automatically without human intervention. What is more important further research includes machine learning model enhancements. We want to develop methods that will not only detect failures or provide additional information compared to the on-board computer but can also be used, for example, to assess the driver’s driving style, especially from an economic point of view.

## Figures and Tables

**Table 1:** Data sample.

*i*	1	2	3	4	5	6	7	8	9	10	11	12	13
$x_{i}$	6.3	7.0	6.7	6.7	6.7	7.0	5.6	6.3	7.8	5.1	6.3	6.2	6.2
$p(x_{i})$	0.17	0.16	0.18	0.18	0.18	0.16	0.10	0.17	0.07	0.04	0.17	0.15	0.15
*i*	14	15	16	17	18	19	20	21	22	23	24	25	26
$x_{i}$	6.2	5.6	6.3	6.7	7.0	5.6	7.8	6.7	6.2	6.7	6.7	7.0	6.3
$p(x_{i})$	0.15	0.10	0.17	0.18	0.16	0.10	0.07	0.18	0.15	0.18	0.18	0.16	0.17
*i*	27	28	29	30	31	32	33	34	35	36	37	38	39
$x_{i}$	7.8	7.8	7.8	7.0	6.7	7.0	6.2	6.7	7.0	5.0	6.2	6.3	6.2
$p(x_{i})$	0.07	0.07	0.07	0.16	0.18	0.16	0.15	0.18	0.16	0.01	0.15	0.17	0.15
*i*	40	41	42	43	44	45	46	47	48	49	50		
$x_{i}$	7.4	7.4	8.0	7.0	6.7	7.0	7.0	6.3	7.0	6.7	6.2		
$p(x_{i})$	0.10	0.10	0.02	0.16	0.18	0.16	0.16	0.17	0.16	0.18	0.15		

**Table 2:** Probability distribution.

$x_{i}$	5.0	5.1	5.6	6.2	6.3	6.7	7.0	7.4	7.8	8.0
$p(x_{i})$	0.01	0.04	0.10	0.15	0.17	0.18	0.16	0.10	0.07	0.02

**Table 3:** CSV headers with descriptions.

Parameter	Description
GPS Time	time based on GPS satellites
Device Time	smartphone based time
Longitude	geographic coordinate
Latitude	geographic coordinate
GPS Speed (Meters/second)	speed measured based on GPS satellites
Horizontal Dilution of Precision	horizontal dilution of precision
Altitude	altitude measured based on GPS satellites
Bearing	magnetic bearing based on smartphone magnetic field sensor
G(x)	acceleration in *x* axis
G(y)	acceleration in *y* axis
G(z)	acceleration in *z* axis
G(calibrated)	combined G value
EGR Error (%)	calculated EGR error
Barometric pressure (from vehicle) (psi)	barometric pressure
Intake Manifold Pressure (psi)	intake pressure
Fuel Rail Pressure(psi)	fuel rail pressure
Run time since engine start (s)	run time since engine start
Trip time (whilst stationary) (s)	trip time while stationary
Trip time (whilst moving) (s)	trip time while moving
Trip Time (Since journey start) (s)	total trip time
GPS Bearing (${}^{{}^{\circ}}$)	bearing based on GPS satellites
Timing Advance (${}^{{}^{\circ}}$)	timing advance
Litres Per 100 Kilometer (Instant) (l/100 km)	instant fuel consumption
Horsepower (At the wheels) (hp)	power delivery in horsepower
Engine kW (At the wheels) (kW)	power delivery in kilowatts
Torque(Nm)	torque in Newton meters
Voltage (OBD Adapter) (V)	measured voltage on OBD adapter
Voltage (Control Module) (V)	measured voltage on control module
Engine Load(%)	engine load
Engine RPM (rpm)	engine revolutions per minute
Distance traveled with MIL/CEL lit (km)	distance traveled with check engine light
Distance traveled since codes cleared (km)	how much kilometers were driven since error codes were cleared
Percentage of City driving (%)	percentage of city driving
Percentage of Highway driving (%)	percentage of highway driving
Percentage of Idle driving (%)	percentage of idle driving
Trip Distance (km)	distance since start for single measurement
Trip distance (stored in vehicle profile) (km)	application specific value
Mass Air Flow Rate (g/s)	amount of air
Speed (OBD) (km/h)	speed measured by car
EGR Commanded (%)	commanded EGR
Ambient air temp (°C)	ambient air temperature
Intake Air Temperature (°C)	intake air temperature
Engine Coolant Temperature (°C)	engine coolant temperature
Turbo Boost & Vacuum Gauge (psi)	turbocharger boost
Trip average KPL(kpl)	average kilometers traveled using 1 L of fuel
Trip average Liters/100 KM (L/100 km)	average fuel burnt in liters per 100 km

**Table 4:** Probabilities in reference model.

Temperature	Value
68	0.004653044709690472
69	0.003641513251062108
70	0.0020230629172567267
71	0.0020230629172567267
72	0.0010115314586283633
73	0.00303459437588509
74	0.0014161440420797087
75	0.0016184503338053813
76	0.0020230629172567267
77	0.0038438195427877805
78	0.02650212421606312
79	0.003641513251062108
80	0.0016184503338053813
81	0.0050576572931418165
82	0.01658911592150516
84	0.0414727898037629
85	0.06048958122597613
86	0.0540157798907546
87	0.0776856160226583
88	0.08436172364960551
89	0.10621080315597815
91	0.20149706655876998
90	0.10095083957111066
92	0.07647177827230427
83	0.036819745094072424
93	0.035201294760267045
94	0.030952862634027918
95	0.01294760267044305
96	0.002225369208982399

**Table 5:** Selected α for each calculated entropy type.

Entropy	Positive α Value	Negative α Value
Renyi	4	-2.5
Tsallis	4	-2.5

**Table 6:** Created models based on specific attributes.

Model Name	Coolant Temperature	Renji Entropy	Tsallis Entropy	Mean	Median
**Model A**	x	-	-	-	-
**Model B**	-	-	x	-	-
**Model C**	-	x	x	x	x
